# Hutchinson-Gilford progeria syndrome alters the endothelial genetic response to laminar shear stress

**DOI:** 10.3389/fphys.2025.1599339

**Published:** 2026-02-24

**Authors:** Crystal C. Kennedy, Jonathan L. Carter, George A. Truskey

**Affiliations:** 1 University Program in Genetics and Genomics, Duke University, Durham, NC, United States; 2 Department of Biomedical Engineering, Duke University, Durham, NC, United States

**Keywords:** HGPS: Hutchinson-Gilford progeria syndrome, endothelium, shear stress (fluid), atherosclerosis, LGALS3

## Abstract

**Introduction:**

Hutchinson-Gilford Progeria Syndrome (HGPS) is a fatal, accelerated-aging disease caused by a mutation in the nuclear envelope protein Lamin A. The resulting mutant protein, progerin, accumulates on the nuclear envelope, causing nuclear blebbing, altered gene expression, and other cellular defects. The primary pathology of HGPS is atherosclerosis, leading to stroke or heart attack. Given that atherosclerosis generally begins with endothelial dysfunction, we examined whether the HGPS endothelium has an altered genetic response to shear stress, contributing to atherogenesis.

**Methods:**

We exposed HGPS and healthy iPSC-derived endothelial cells (viECs) to steady laminar shear stress at 12 dyn/cm^2^ in a parallel-plate flow chamber. We examined morphology changes, differential gene expression (DE) via RNA-seq, and Gene Set Enrichment Analysis (GSEA) after 24 h.

**Results:**

Elongation after flow is impaired in HGPS viECs compared with healthy viECs. DE analysis showed fewer significant DE genes and a lower magnitude of gene expression change after flow in HGPS compared with healthy viECs. GSEA identified differences in the gene sets altered by flow-induced DE, including Cholesterol Homeostasis, which was overrepresented in HGPS viECs. LGALS3, encoding the atherosclerosis marker galectin-3, was a main driver of this overrepresentation. RT-PCR confirmed LGALS3 is robustly upregulated in HGPS viECs compared with healthy viECs after flow. Treatment with an adenine base editor correcting the HGPS mutation restored LGALS3 expression to healthy levels.

**Conclusion:**

These observations indicate that HGPS ECs have an aberrant molecular response to atheroprotective shear stress, including impaired elongation and upregulation of the pro-inflammatory gene LGALS3, which contributes to atherogenesis in HGPS patients.

## 1 Introduction

Hutchinson-Gilford Progeria Syndrome (HGPS) is a rare and fatal accelerated-aging disease with cases currently reported for 154 children worldwide (Progeria Research Foundation, 2024). Symptoms first occur in toddlers and include signs of rapid aging such as sclerotic skin, alopecia, and subcutaneous fat loss (DeBusk, 1972; Eriksson et al., 2003). The average lifespan of an HGPS patient is 14.5 years (Progeria Research Foundation, 2024; Gordon et al., 2003), with death due to cardiovascular disease (CVD) in over 90% of cases (Eriksson et al., 2003).

HGPS is caused by a *de novo*, heterozygous point mutation in the *LMNA* gene, which results in the translation of a truncated and farnesylated protein called progerin (Eriksson et al., 2003; Gordon et al., 2003). *LMNA* is located on the q arm of chromosome 1 and is responsible for the expression of two nuclear envelope filament proteins, Lamin A in exons 1-12 and Lamin C in exons 1-10^3^. Lamin A is first transcribed as pre-lamin A, containing a -CAAX motif with a farnesyl group attached at the cysteine (Davies et al., 2009). Post-translational modifications of pre-lamin A result in removal of the farnesyl group before formation of the mature Lamin A product (Eriksson et al., 2003; Davies et al., 2009). In classic HGPS cases, a C to T mutation in exon 11 of *LMNA* causes aberrant splicing in the pre-lamin A transcript, leading to the progerin protein which is truncated by 50 amino acids and retains the farnesyl group (Eriksson et al., 2003; Gordon et al., 2003). Progerin accumulates on the nuclear envelope, leading to abnormal nuclear envelope structure (Eriksson et al., 2003) and other deleterious cellular consequences such as increased oxidative stress, DNA damage and cellular senescence (Guenantin et al., 2014).

HGPS patients develop severe atherosclerosis, which is the main contributor to HGPS fatality via stroke and heart attack. Atherosclerosis in non-HGPS individuals begins when a dysfunctional endothelium becomes infiltrated with low-density lipoprotein, leading to monocyte accumulation and subsequent fatty streak formation (Rafieian-Kopaei et al., 2014; Danielsson et al., 2022). Risk factors for atherosclerosis include aging, smoking, high cholesterol, obesity and hypertension (Rafieian-Kopaei et al., 2014). HGPS patients, however, lack these risk factors and develop atherosclerosis more rapidly than typical CVD patients (Gimbrone and Garcia-Cardena, 2016). Common features of atherosclerosis in HGPS and non-HGPS patients include occlusion of the vascular lumen and calcification of vessel walls (Gimbrone and Garcia-Cardena, 2016; Davies, 2009). Pulse wave velocity readings are comparable between children with HGPS and older CVD patients (Gimbrone and Garcia-Cardena, 2016), suggesting vessel stiffening. Conversely, vasculature in HGPS patients contain higher levels of progerin, loss of medial smooth muscle cells, pronounced adventitial thickening and increased calcification compared with that of general CVD patients (Olive et al., 2010).

Despite endothelial dysfunction being a typical early event in atherogenesis, the endothelial lining has long been overlooked in characterizing the effects of HGPS on vasculature due to low expression of Lamin A (Olive et al., 2010; Rafieian-Kopaei et al., 2014; Gimbrone and Garcia-Cardena, 2016). Endothelial cells (ECs) are mechanosensitive, exhibiting physiological and molecular responses to shear stress—the force exerted on them by blood flow. In conditions of laminar shear stress, ECs elongate themselves parallel to the direction of flow and increase transcription of antioxidant and anti-inflammatory genes, creating an atheroprotective phenotype (Traub and Berk, 1998; Gimbrone and Garcia-Cardena, 2016; Davies, 2009). Low shear stress and oscillatory flow experienced at branched areas of the vasculature have the opposite effect, promoting an atheroprone environment consisting of increased monocyte adhesion and inflammatory markers (Gimbrone and Garcia-Cardena, 2016).

In more recent studies of progerin’s effect on ECs, transgenic mice with endothelial-specific progerin overexpression showed a reduced endothelial shear stress response, a decrease in production of the vasodilator and antioxidant nitric oxide (NO), and fibrosis of the heart and arteries (Osmanagic-Myers et al., 2019). Gene expression of HGPS endothelium has also been examined, with dysregulation found in genes associated with DNA damage repair and EC identity (Mojiri et al., 2021). In studies using primary ECs with exogenously expressed progerin or a ZMPSTE24 knockdown to model HGPS endothelium, laminar shear stress induces cell loss and nuclear abnormalities, indicating maladaptation to atheroprotective shear stress (Danielsson et al., 2022). We have previously found that ECs generated from human HGPS iPSCs show an impairment in flow alignment, an abnormal downregulation of endothelial nitric oxide synthase (eNOS) after flow, and expression of leukocyte adhesion molecules (Atchison et al., 2020). Additionally, tissue-engineered blood vessels fabricated with HGPS endothelium display reduced vasoactivity (Atchison et al., 2020). These defects in HGPS EC function are alleviated upon treatment with the HGPS drugs lonafarnib and everolimus (Abutaleb et al., 2023). Taken together, these studies suggest a role in endothelial-specific dysfunction in HGPS atherosclerosis.

While gene expression of static HGPS ECs (Mojiri et al., 2021) and adaptation of progerin-expressing ECs under shear stress (Danielsson et al., 2022) have been examined separately, the global genetic response to shear stress in HGPS ECs has not been assessed. We hypothesize that HGPS ECs exhibit an impairment in their genetic response to laminar shear stress, promoting an atheroprone phenotype instead of an atheroprotective one. Thus, we examined whether differential gene expression differs between HGPS ECs and healthy ECs under shear stress, and whether this difference in response can contribute to disease progression. To do this, iPSCs from HGPS and healthy donors were differentiated to ECs (viECs) and subjected to physiologically relevant shear stress for 24 h. RNAseq analysis was then performed to determine pathways and genes that show differing flow responses between healthy and HGPS phenotypes.

## 2 Materials and methods

### 2.1 Human umbilical vein endothelial cell (HUVEC) culture

HUVECs were provided by Clonetics and were cultured on T-75 flasks in 15 mL Endothelial Cell Growth Media (Cell Applications). Media was changed every other day following a PBS rinse.

### 2.2 Differentiation of iPSCs to endothelial cells (viECs) and viEC culture

Three healthy (168CL2, 168CL3, and 0901B) and 2 HGPS (0031D and 167CL2) induced pluripotent stem cells were provided by the Progeria Research Foundation. Once confluent, iPSCs were passaged on to Matrigel-coated T-75 flasks using Accutase (Stemcell Technologies) and fed with mTeSR+ media supplemented with 10 µM of the Rho-kinase inhibitor Y27632 (Fischer Scientific) on Day 0. Cells then underwent a 7-day differentiation protocol as outlined by Pastch et al. (Patsch et al., 2015) and modified by Atchison et al. (Atchison et al., 2020). Briefly, mesoderm induction was promoted on Day 1 with the addition of N2B27 (Neurobasal media, DMEM/F12, N2 supplement, B27 supplement; ThermoFisher) media supplemented with 8 µM CHIR99021 (Cayman Chemical) and 25 ng/mL hBMP4 (PeproTech) and incubated for 3 days. For endothelial induction, Stempro-34 serum free media (ThermoFisher) supplemented with 1% GlutaMax (ThermoFisher), 200 ng/mL VEGF (GenScript) and 2 µM forskolin (Selleck Chemicals) was added on day 4. On day 5 conditioned media was collected and endothelium-inducing media was replenished. This was repeated on day 6. On day 7, conditioned media was collected for the third time and EC selection was performed with MACS MicroBead Technology (Miltenyi Biotec) using CD31^+^ (PECAM1) and CD144+ (VE-cadherin) magnetic beads. Selected viECs were then plated on T-75 flasks coated with rat tail collagen-1 (Corning) and fed with conditioned media then viEC media (StemPro-34 with 1% GlutaMax, 50 ng/μL VEGF, 10% HI-FBS (ThermoFisher) and 2 μg/mL heparin (Sigma)) every 2 days until 70% confluent. ECs were verified previously by immunostaining for vWF (Abcam, 1:200) (Abutaleb and Truskey, 2021) and progerin in HGPS viECs by Western blot (Atchison et al., 2020).

### 2.3 Laminar flow experiments

Flow experiments were performed as described previously (Atchison et al., 2020; Abutaleb et al., 2023). Superfrost microscope slides (VWR) were first sterilized by wiping with 70% ethanol and placing under UV light with the intended cell culture surface facing upward for 30 min. The clear part of the slide was outlined with a hydrophobic pen and coated with rat-tail collagen 1. Confluent viECs in a T-75 flask were passaged using Accutase, resuspended in 3 mL viEC media, and evenly distributed across three collagen-coated microscope slides in a square petri dish. Slides were overseeded with ∼1.5million cells each, so that they were confluent immediately. The cell suspension was left untouched for 30 min so that cells adhered to the surface. Then, 12 mL viEC media were added to the square dish to cover all slides. Cells were left in an incubator at 37°C and 5% CO_2_ for at least 24 h before a flow experiment. To conduct flow experiments, a slide is placed in a custom-built parallel plate flow chamber (Abutaleb and Truskey, 2021) and connected to a media reservoir, a pulse dampener, and a Masterflex digital modular drive pump (Cole-Parmer). The flow set-up is placed in an incubator at 37°C and 5% CO_2_. The speed of the pump is ramped up in increments to gradually increase the shear stress applied; approximately 2 dyn/cm^2^ were applied for 15 min, 4 dyn/cm^2^ for 45 min, 7 dyn/cm^2^ for 1 h, and finally 12 dyn/cm^3^ for 24 h. For each flow experiment a slide was left in the static condition.

### 2.4 Phase contrast imaging and quantification of EC orientation and elongation

Three phase contrast images were taken of each slide under flow and its corresponding static control slide. Alignment and roundness were measured for each picture in ImageJ. A grid was placed over each picture, and cells at or near the meeting points of the grid were outlined to reduce bias in selecting cells. Fifty cells were outlined per image, and their angle and roundness measured via ImageJ software. Angles were transformed to represent the angle from the flow direction. The roundness is defined as 4area/π (major axis) (DeBusk, 1972), then angle and roundness values were averaged per image in Excel. An average angle and roundness value was then calculated for each slide, using averages of the three images of that slide. Angularity and roundness were compared between the static controls and cells under flow for each experiment in each line, using R software.

### 2.5 RNA isolation

RNA isolation was performed using the Qiagen RNEasy kit following manufacturer protocols. Resulting RNA was quality checked and quantified using the NanoDrop and stored at −80°C prior to sequencing. Quality scores are included in Supplementary Table S1.

### 2.6 RNA sequencing and analysis

Library preparation and RNA sequencing was carried out by the Duke Genomic and Computational Biology core. Sequencing was single ended and 30 bp in length for experiment 1, and pair-ended and 50 bp in length for experiment 2. Quality scores are listed in Supplementary Tables S1, S2 respectively. Fastq files were processed with a bash script to trim adapters and low-quality reads (TrimGalore! (Krueger, 2025)), check quality of files (FastQC (Andrews, 2025)), align to the human genome hg38 and quantify read counts (STAR (Dobin et al., 2013)). RSEM (Li and Dewey, 2011) was used to verify the truncated progerin transcript in HGPS samples. Read count data was processed using DESeq2 (Love et al., 2014; Zhu et al., 2019) in R to quantify differential expression and pathway analysis was done using GSEA (Subramanian et al., 2005) software.

### 2.7 Lentiviral transduction of HGPS iPSCs

100,000 iPSCs were seeded into one well of a 6-well plate with 0.002 (v/v) lentivirus delivering ABE7.10max-VRQR (Koblan et al., 2021). The lentivirus was removed after 24 h, and the media was refreshed. Two days later, 1 μg/mL puromycin was added and replenished daily for 4 days. Selected iPSCs were then expanded and differentiated normally to viECs. Editing was verified by Sanger Sequencing.

### 2.8 RT-PCR

Primers sequences were obtained via PrimerBank (Wang et al., 2012) for the following genes: NOSTRIN, GPC1, LGALS3, and ADAMTS1 and ordered through Integrated DNA Technologies. GAPDH from Real-Time Primers was used as the housekeeping gene in all analysis. Primer sequences are listed in Supplementary Table S3. Flow experiments were repeated and RNA isolated and quantified, as described above. The LunaScript RT SuperMix Kit from New England Biolabs was used to convert RNA samples to cDNA, and RT-PCR was done using the iQ SYBR Green Supermix (BioRad) and the CFX Connect Real-Time PCR Detection System (Bio-Rad). Analysis of RT-PCR data was performed in Microsoft Excel using the 2^−∆∆Cq^ method (Livak and Schmittgen, 2001) and global normalization.

### 2.9 Statistical methods

Student’s t-test and Analysis of Variance ANOVA with a Tukey *post hoc* test and plots were generated in JMP and GraphPad Prism software respectively.

## 3 Results

### 3.1 HGPS viECs show impaired alignment to laminar shear stress

We first analyzed the morphological response of HGPS and healthy viECs to shear stress. We differentiated iPSCs from three healthy cell lines (168CL2, 168CL3, and 0901B) and 2 HGPS cell lines (0031D and 167CL2) into endothelial cells, referred to as viECs. Both HGPS donors displayed the classical mutation for progerin, c.1824C>T. Cells were seeded onto slides so that they were confluent upon attachment. It was observed that confluent HGPS viECs had the appearance of lower density than healthy viECs despite overseeding, and leaving them in culture resulted in cell loss rather than cell doubling at that stage. Cells were exposed to laminar flow at a shear stress of 12 dyn/cm^2^ using a parallel-plate flow chamber. After 24 h, viECs were imaged and measured for orientation and roundness, with viECs exposed to shear stress being compared with their static controls.

**FIGURE 1:**
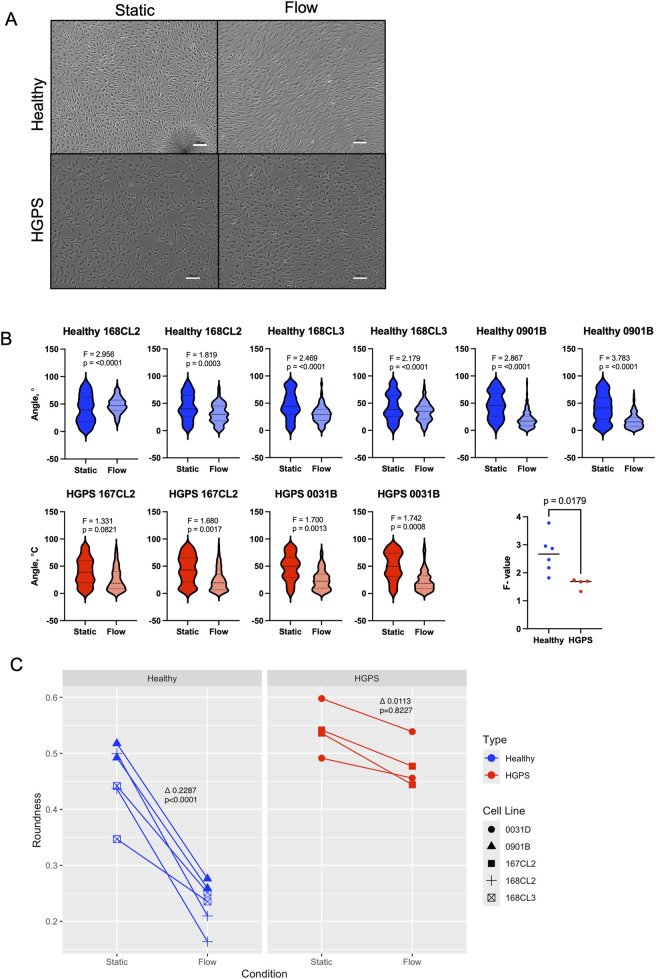
HGPS viECs show impaired alignment in response to laminar shear stress. **(A)** Representative images of healthy and HGPS viECs in static conditions (left) and after 24 h of shear stress at 12 dyn/cm^2^ (right). Scale bars: 100 µm. **(B)** Violin plots displaying range of cell orientation angles before and after 24 h laminar shear stress in individual experiments. F-test performed per experiment show that healthy viECs had a significantly smaller variation in angles after flow. N = 150, degrees of freedom (df) = 149, α = 0.05. **(C)** Cell roundness measured in static conditions and after 24 h of shear stress at 12 dyn/cm^2^ (N = 150). Delta values indicate difference in average roundness between conditions.

Visually, elongation and alignment of cells were more pronounced in healthy viECs compared with HGPS viECs (Figure 1A). After flow the range of cell orientation angles was decreased more significantly in healthy viECs than in HGPS viECs (Figure 1B), illustrating more pronounced cell alignment in healthy viECs. This is highlighted by the F-statistic that indicates whether variance in angle values changes significantly between cells under static and flow conditions. This F-value is the ratio of variance between group means (that is, cell orientation angles in static and flow conditions) to the variance within groups, with a higher F-value indicating a larger difference between conditions relative to within-group variation. Healthy viECs display significantly higher F-values than HGPS viECs (n = 150, alpha = 0.05, df = 149). Thus, the variance in the angle of alignment decreases more significantly after flow in healthy viECs compared with HGPS viECs, indicating more pronounced directionality in response to shear stress of healthy viECs (Figure 1B). Cell elongation was also quantified in terms of roundness, with a roundness value closer to 0 representing a more elongated shape. HGPS viECs underwent a non-significant change in cell elongation after 24 h of shear stress, while healthy viECs (p < 0.0001) displayed a significant change in roundness (Figure 1C). The delta value (Δ) is the mathematical difference in average roundness values between static cells and flow-exposed cells for each cell phenotype. These observations show impaired elongation in response to shear stress and indicate decreased endothelial mechanosensitivity to laminar shear stress in HGPS viECs.

### 3.2 Healthy viECs and HUVECs show similar genetic response to shear stress

To establish whether the genetic response to shear stress in healthy viECs was similar to that of primary ECs, an RNAseq study was carried out comparing HUVECs and healthy viECs under static and flow conditions. RNA was isolated from HGPS and healthy viECs exposed to 24 h of 12 dyn/cm^2^ shear stress and their static controls. All RNA was of high integrity and sequence quality (Supplementary Table S1). Consistent with prior reports that primary HUVECs and viECs display differing gene signatures related to their primary versus iPS-derived natures (Wiseman et al., 2019; Rieck et al., 2024), we observed differences between the HUVECs and viECs in a PCA plot (Supplementary Figure S1A). However healthy viECs and HUVECs showed a similar gene expression change associated with 24 h shear stress (Supplementary Figure S1B), and a significantly correlated trend in the differential expression of known shear-sensitive genes (Supplementary Figure S1C).

### 3.3 HGPS viECs show repressed gene expression in comparison with healthy viECs

**FIGURE 2:**
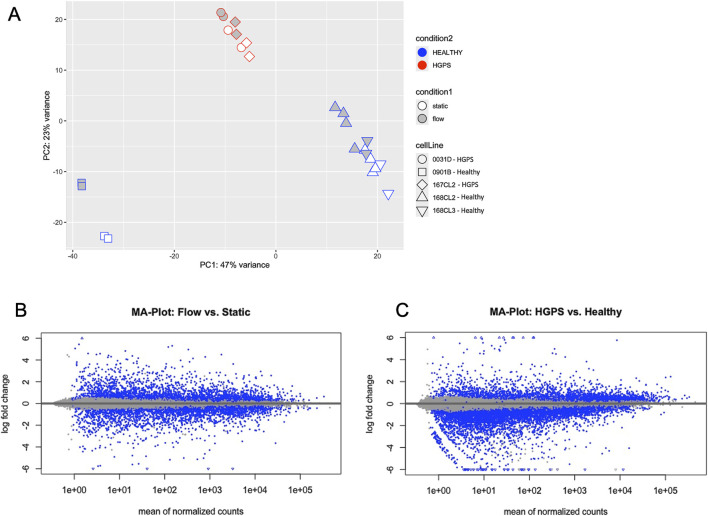
HGPS viECs show repressed gene expression response to flow in comparison with healthy viECs. **(A)** Principal components analysis of RNA sequences for healthy and HGPS viECs under flow and static conditions. HGPS samples cluster away from healthy samples, separated primarily by both PC1. The effect of flow was captured by differences in PC2. **(B, C)** MA Plots showing mean of normalized counts vs. Log_2_Fold Change of genes in combined HGPS and healthy RNA samples. Blue dots represent differentially expressed genes, with blue arrows indicating genes that lay beyond the y-axis limit. **(B)** Counts are normalized to static samples. **(C)** Counts are normalized to healthy samples.

Given that HGPS viECs showed impaired morphological changes and decreased NO release after flow exposure (Atchison et al., 2020; Gete et al., 2021), we hypothesized that further global gene expression differences may exist in HGPS viECs in response to physiologically relevant laminar shear stress. We performed RNA sequencing and differential expression analysis of healthy and HGPS viECs under static and flow conditions. Principal components analysis (Figure 2A) shows differences between the cell lines along PC1. Both healthy and HGPS phenotypes show a genetic response to shear stress, with flow samples shifting upward away from static samples in PC2. Of note is that this shift is less pronounced in HGPS samples, potentially indicating a less robust flow response. Additionally, HGPS viECs show a large deviation from healthy viECs along PC2. This indicates that while both phenotypes exhibit a shear stress response, there is a difference in the overall gene expression pattern of HGPS viECs compared with healthy viECs.

**FIGURE 3:**
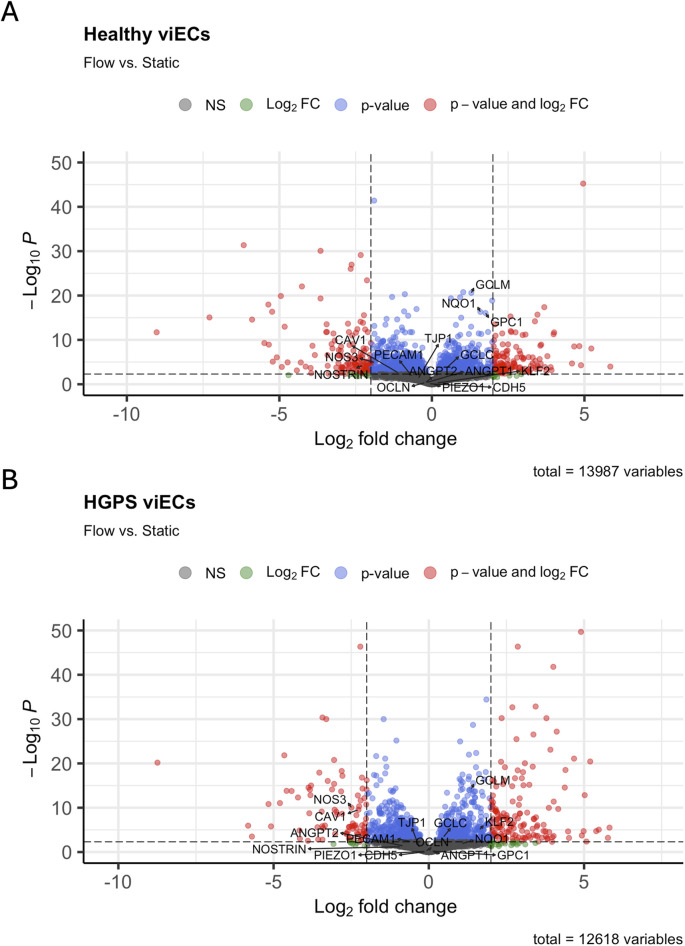
Volcano plots displaying differential expression of genes in flow vs. static conditions in **(A)** healthy and **(B)** HGPS viECs. EC markers and genes of interest are labeled.

MA plots are used to visualize overall differential expression between two groups (McDermaid et al., 2019). Approximate Posterior Estimation for generalized linear model (*apeglm*) was applied to normalize counts for log_2_ fold change (L2FC) shrinkage (Zhu et al., 2019). MA plots comparing flow and static conditions across healthy and HGPS samples show a generally even distribution of differentially expressed genes across the y-axis, indicated by the dispersion of blue points evenly across the plot. Thus, this demonstrates that gene expression is altered by flow in both healthy and HGPS phenotypes (Figure 2B; Supplementary Figure S2), and is also shown demonstrated in volcano plots (Figure 3). However, when HGPS samples are analyzed against healthy samples, gene expression points shift downward on the y-axis of the MA plot (Figure 2C), demonstrating a diminished level of gene expression in HGPS viECs. Taken together, these analyses show that general gene expression in HGPS viECs is altered and diminished in comparison to healthy viECs.

### 3.4 HGPS viECs show gene expression differences in shear stress response compared with healthy viECs

**FIGURE 4:**
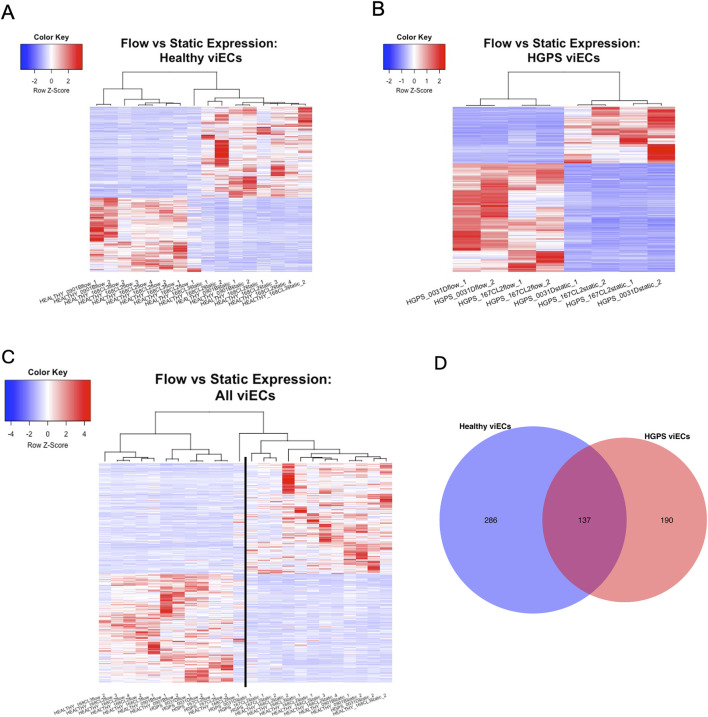
**(A–C)** Heatmaps showing response to shear stress in healthy and HGPS viECs. Each line represents a gene with a L_2_FC of ≥ |2| and adjusted p-value of ≤0.05. **(C)** Flow samples separate to the left of the line while static samples separate to the right. Healthy samples congregate to the ends of the heatmap, showing a larger magnitude of differential expression between healthy flow and static samples. **(D)** The number of significant differentially expressed genes in healthy and HGPS viECs. Normalized counts were shrunk using the *apeglm* method.

To look more closely at the pattern of differential expression (DE), heatmaps and volcano plots were generated to visualize flow response in both healthy and HGPS viECs. Genes with a L2FC of $\geq \left|2\right|$ and an adjusted p-value of ≤0.05 were selected. Volcano plots (Figure 3) and heatmaps (Figures 4A, B) for each phenotype show that both HGPS and healthy viECs experience DE in response to shear stress. One outlier was found among the healthy experiments, with a shear stress sample from the healthy 168CL2 cell line resembling healthy static samples.

A heatmap comparing all samples shows healthy viECs display a larger magnitude of differential expression in response to shear stress, with flow samples congregating on the far left of the heatmap and static samples on the far right. HGPS samples also separate by flow or static condition but are gathered closer to the center (Figure 4C), illustrating that shear stress invokes a more significant response in healthy viECs. Results also show a smaller number of significant differentially expressed genes in the HGPS flow response. Healthy viECs show differential expression of 286 unique genes, in contrast to 190 in HGPS viECs. There is an overlap of 137 differentially expressed genes between both phenotypes (Figure 4D). Taken together, analysis shows diminished differential expression in response to laminar shear stress in HGPS viECs.

### 3.5 Gene sets affected by shear stress differ between HGPS and healthy viECs

Gene Set Enrichment Analysis (GSEA) was performed on the RNAseq data to determine potential pathways that are affected by shear stress in healthy and HGPS viECs, and to assess any differences in this response between these two phenotypes. Differences were found both in significantly overrepresented and underrepresented gene sets when shear stress is applied to healthy or HGPS viECs (Table 1). One such set representing the Epithelial-to-Mesenchymal Transition (EMT) shows a higher normalized enrichment score (NES) and more leading-edge genes in healthy viECs (NES 2.092) than in HGPS viECs (NES 1.54), indicating that EMT-associated genes are activated more widely in healthy viECs than in HGPS viECs in response to laminar shear stress (Table 1; Supplementary Figure S3A). Alternately, some sets such as those representing Cholesterol Homeostasis, Androgen Response and KRAS Signaling Downregulation, show more representation in the HGPS viECs’ flow response indicated by higher NES values and lower false discovery rates (Table 1; Supplementary Figures S3B–D). Underrepresented gene sets include Interferon-*α* Response and Interferon-*γ* Response sets, which show significantly lower NES values in healthy viECs compared with HGPS viECs (Table 1), indicating a decreased inflammatory and immune response in healthy cells. Conversely, the G2M Checkpoint set shows more significant underrepresentation in HGPS viECs, suggesting decreased recognition of DNA damage in HGPS. The MYC Targets V1 gene set returned no results for healthy cells, while showing underrepresentation in HGPS viECs.

**TABLE 1:** GSEA of viECs in response to shear stress.

Gene set	Healthy			HGPS		
	Normalized enrichment score	FDR	# Leading edge genes	Normalized enrichment score	FDR	# Leading edge genes
Epithelial-mesenchymal transition	2.092	0.000%	65	1.54	3.700%	56
Cholesterol homeostasis	1.668	1.200%	35	2.14	0.000%	32
Androgen response	1.559	2.100%	24	1.849	0.200%	24
KRAS signaling downregulation	1.068	37.700%	31	1.58	3.600%	29
G2M checkpoint	−1.846	0.084%	119	−2.31	0.000%	119
Interferon alpha response	−2.328	0.000%	45	−1.55	1.000%	32
Interferon gamma response	−2.065	0.000%	64	−1.742	0.300%	64
MYC targets V1	NA	—	—	−1.864	0.072%	102

Blue rows indicate gene sets that are more significant for healthy viECs, red rows indicate gene sets that are more significant for HGPS viECs.

### 3.6 LGALS3 shows robust upregulation in HGPS viECs in response to shear stress

**FIGURE 5:**
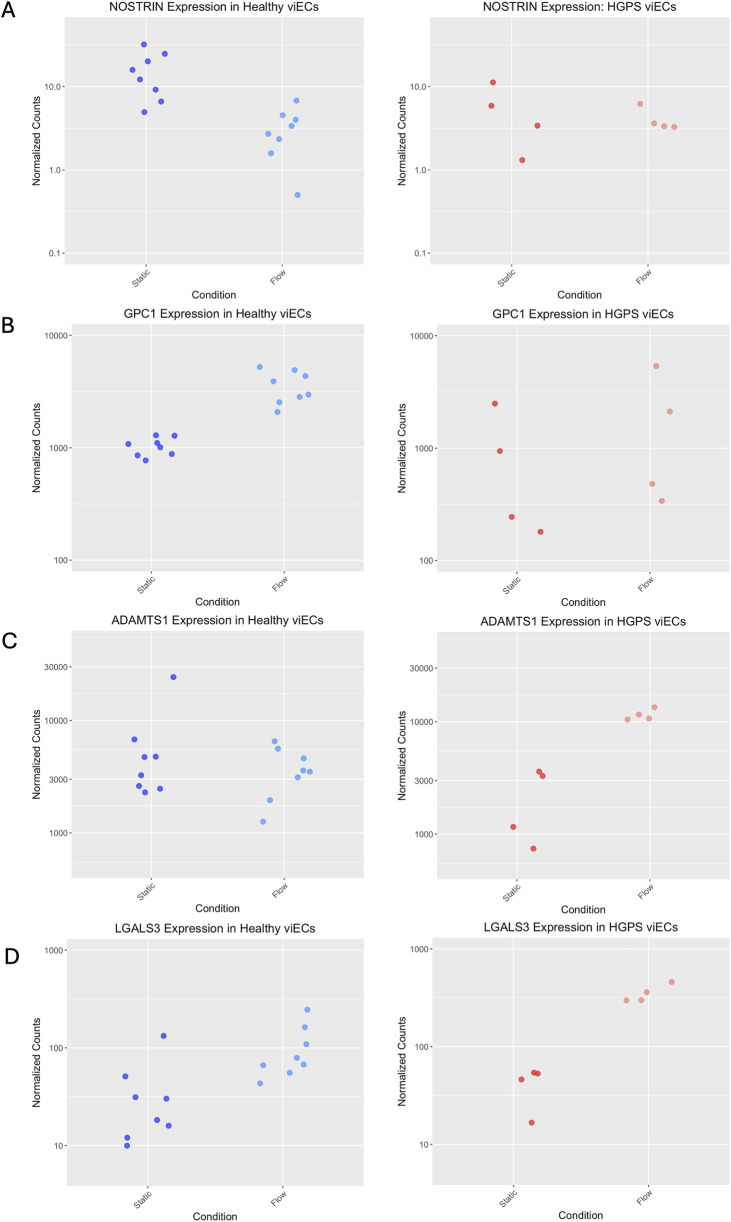
Plots showing read counts of four differentially expressed genes between healthy and HGPS viECs before and after flow exposure, **(A)** NOSTRIN, **(B)** GPC1, **(C)** ADAMTS1, and **(D)** LGALS3. Note that Y-axes are on a log10 scale.

Genes of interest from the differential expression analysis were chosen for RT-PCR validation based on significant differences in their flow response between each phenotype. These were NOSTRIN, GPC1 from the EMT gene set, ADAMTS1 from the Androgen Response gene set, and LGALS3 from the Cholesterol Homeostasis gene set. NOSTRIN is a gene responsible for sequestering NO and works antagonistically with eNOS (Zimmermann et al., 2002). RNAseq data showed NOSTRIN as downregulated robustly under flow in healthy viECs as expected, but unaffected by flow in HGPS viECs. (Table 2; Figure 5A). GPC1, encoding a glycocalyx mechanosensing molecule (Bartosch et al., 2021), stood out as a major driver of difference in the EMT gene set representation between the healthy and HGPS viEC flow response (Supplementary Figure S3A), showing significant upregulation under flow in healthy viECs and no change in HGPS viECs (Table 2; Figure 5B). On the other hand, ADAMTS1, a gene implicated in myocardial infarction (Pehlivan et al., 2016), showed significant upregulation in HGPS viECs (Table 2; Figure 5C) and is high on the list of leading-edge genes for the Androgen Response gene set in HGPS (Supplementary Figure S3D). No change in expression was detected in the healthy viEC flow response. Our fourth gene of interest, LGALS3 encodes the atherosclerosis marker galectin-3 and is upregulated in both healthy and HGPS viECs in response to shear stress (Table 2; Figure 5D). However, the upregulation in HGPS cells was more robust, placing LGALS3 at the top of the Cholesterol Homeostasis gene set for HGPS viECs and contributing to its overrepresentation (Supplementary Figure S3B).

**TABLE 2:** Log_2_ fold change and adjusted p-values of genes of interest from differential gene expression analysis.

Gene symbol	Healthy		HGPS	
	Log_2_ FoldChange	p-adj	Log_2_ FoldChange	p-adj
NOSTRIN	−2.260	5.48E-05	−0.893	6.55E-02
GPC1	1.758	8.64E-17	0.089	2.85E-01
ADAMTS1	−0.255	3.43E-01	2.219	8.62E-08
LGALS3	1.035	3.32E-02	2.996	2.19E-17

To validate the RNAseq results of these select genes of interest, flow experiments were repeated for RT-PCR. Additionally, to determine whether correcting the causative HGPS mutation restores healthy expression levels, we obtained HGPS viECs that were treated with an ABE7.10 CRISPR-Cas9 base editor to correct the C>T mutation that results in progerin expression. Cells were placed under 24 h shear stress as previously described, RNA isolated and RT-PCR performed on the above listed genes.

**FIGURE 6:**
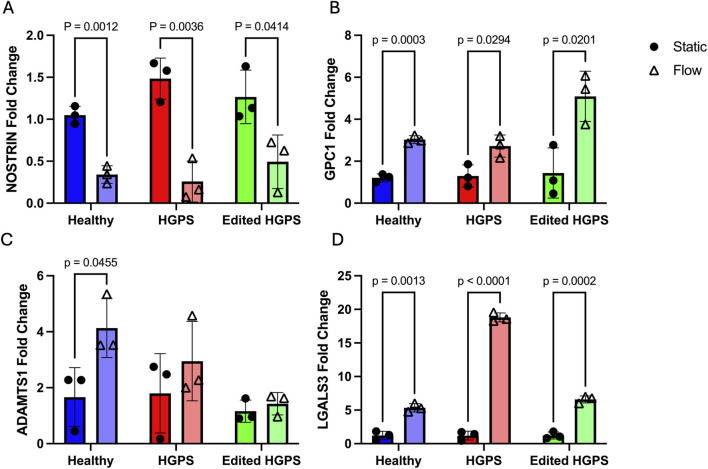
RT-PCR of mRNA expression of four differentially expressed genes between healthy and HGPS viECs before and after flow exposure, **(A)** NOSTRIN, **(B)** GPC1, **(C)** ADAMTS1, and **(D)** LGALS3. N = 3 per condition. Data analyzed using a paired t-test between static and flow conditions.

For each cell type, the flow condition was normalized to its corresponding static condition. NOSTRIN expression was significantly downregulated after flow in both healthy and HGPS viECs (Figure 6A), differing from RNAseq results that showed no change in expression after flow in HGPS viECs. This was also observed in edited HGPS viECs. It is notable that the count values for NOSTRIN in the RNAseq data were fairly low (Figure 5A), indicating a lowly expressed gene which can often make statistics unreliable, thus explaining the discrepancy with RT-PCR. Significant GPC1 upregulation after flow was replicated in healthy cells, and was also observed in HGPS viECs (Figure 6B), unlike RNAseq results that showed no change in HGPS viECs (Figure 5B; Table 2). Interestingly, upregulation of GPC1 with shear stress in HGPS viECs appears to be less pronounced than that of healthy cells, as indicated by the larger p-value. Base editing of HGPS viECs leads to GPC1 upregulation under flow at a higher fold change than in both healthy and HGPS viECs respective to their static controls (Figure 6B). While RNAseq results are not fully replicated in RT-PCR of GPC1 expression, it does indicate a pattern of diminished GPC1 flow response in HGPS ECs. RT-PCR of ADAMTS1 also showed differing results from RNAseq data, with significant upregulation in healthy viECs and a non-significant upward trend in HGPS viECs after shear stress (Figure 6C); whereas RNAseq showed no change in expression of ADAMTS1 in healthy viECs and increased expression in HGPS viECs after shear stress (Figure 5C; Table 2). Base-edited HGPS viECs also show no change in ADAMTS1 expression. These discrepancies between RT-PCR and RNAseq observations are predicted to be due to differences in isoforms amplified in either case, and/or low counts and high count variability of genes in the RNAseq data (Everaert et al., 2017).

LGALS3 was the only gene whose RT-PCR results fully replicated the findings of RNAseq analysis. While all cells upregulated LGALS3 after 24 h shear stress, HGPS viECs upregulate LGALS3 approximately 4 times as much as healthy viECs (Figure 6D). In base edited HGPS viECs, LGALS3 upregulation under flow returned to similar levels as seen in healthy viECs, confirming that corrective base editing restores the shear stress response of LGALS3 to healthy levels. Thus, LGALS3 is a shear-sensitive gene that is overexpressed in HGPS endothelium.

## 4 Discussion

These results add to the growing body of evidence that ECs harboring the HGPS point mutation display an aberrant response to otherwise atheroprotective laminar shear stress, corroborating our previous work (Atchison et al., 2020) and expanding upon it by performing an RNAseq analysis. The expected morphological changes due to 12 dyn/cm^2^ shear stress are hampered in HGPS viECs, as indicated by a higher variance in cell orientation angles and higher levels of roundness after flow compared with healthy viECs. HGPS viECs display diminished gene expression compared with healthy viECs, both generally and as a response to shear stress. The altered flow response is emphasized in GSEA, which shows major differences in the gene sets that represent differentially expressed genes. The Cholesterol Homeostasis group is one such gene set, showing significant overrepresentation in HGPS. We identified LGALS3 from this set which appeared as a top leading-edge gene for shear-exposed HGPS viECs. Upregulation of LGALS3 occurs at significantly higher levels in HGPS viECs than in healthy viECs, and this effect was reproduced via RT-PCR in both cell types. Corrective base editing of the HGPS mutation restores LGALS3 upregulation to healthy levels, thereby linking increased LGALS3 expression with progerin expression.

Recent work in HGPS mice showed that eliminating EC-specific progerin expression does not ameliorate the progeroid phenotype, suggesting that progerin-expressing endothelium does not drive the key pathology in HGPS-associated atherosclerosis (Benedicto et al., 2024). Although progerin-expressing ECs alone may not directly induce HGPS pathology, the endothelium does have an important role in the disease. Recent studies show that in the LmnaG609G/G609G mouse model, ECs are more proinflammatory (Barettino et al., 2024) and their pathology is driven, in part, by smooth muscle cells which induce an endothelial-to-mesenchymal transition (Hamczyk et al., 2024). Interestingly, EC specific progerin expression in a mouse model was previously shown sufficient to induce vascular fibrosis, NO decrease and impairment in mechanosensing (Osmanagic-Myers et al., 2019). Additionally, we repeatedly see problematic endothelial behavior in human progerin-expressing ECs (Atchison et al., 2020) including decreased NO release, decreased LDL uptake and signs of senescence (Matrone et al., 2019). It is also established that the HGPS mouse model does not fully mimic HGPS in humans (Bene et al., 2021), and that mouse atherosclerosis operates in a different mechanism compared with humans (Getz and Reardon, 2012). It is therefore still worth assessing the contribution of both vascular cell types to the pathology of HGPS in a human cell system.

HGPS causes nuclear envelope blebbing due to the disruption of the lamin structure caused by progerin (Eriksson et al., 2003). The nuclear envelope is mechanosensitive as is the cellular membrane, and the lamins are involved in sensing and transducing shear stress into multiple cellular processes and pathways (Dasgupta and McCollum, 2019; Aureille et al., 2017). The impaired alignment and elongation of HGPS viECs under shear stress suggests that the compromised lamin structure leads to a disruption in the connection between the nuclear envelope and mechanosensing. This is seen in progerin-expressing primary ECs, which develop nuclear blebbing and experience cell loss after laminar shear stress (Danielsson et al., 2022). It also follows that if mechanosensing is impaired, so are the atheroprotective mechanisms that are generally activated via laminar flow, thus leading to endothelial dysfunction that can aid in atherogenesis. This would explain the observed gene expression differences between healthy and HGPS viECs as shown via PCA and MA plots, as well as the diminished flow response illustrated by further differential expression analysis.

GSEA results identified EMT as a significant gene set represented in the healthy viEC flow response. This is an unexpected find, as the EMT process describes a cellular transition from epithelial to a more migratory mesenchymal identity with increased invasive capability and extracellular matrix deposition, and is associated with fibrosis and cancer processes (Kalluri and Weinberg, 2009). The caveat to GSEA is that while a given set is named for a process broadly associated with the genes within it, these genes often have roles in multiple processes. As such, the Hallmark EMT gene set includes genes that are crucial to endothelial function. For example, CDH2 and TGFb1/2 are activated in multiple pathways in the progression of EMT, and are included in this set for both viEC phenotypes. However, CDH2 (N-cadherin) functions as a junction protein and mediates EC-SMC interactions (Paik et al., 2004; Itoh et al., 2012) and TGFß1/2 signaling is crucial in angiogenesis and promotes vascular stability (Itoh et al., 2012). Similarly, GPC1, one of our genes of interest from the EMT set, is a shear sensitive protein in ECs, comprising part of the glycocalyx and signaling NO production which is crucial for maintaining vascular tone (Bartosch et al., 2021). GPC1 also moonlights as a cancer biomarker, indicating the initiation and progression of EMT in cancer cells (Liu et al., 2021), thus its listing in the EMT set. The function and expression levels of these genes indicates that the EMT gene set representation is best explained by the overlap between genes that are involved in both processes, which explains its representation in both viEC phenotypes. It would also suggest aberrant behavior in the HGPS viECs, given the higher NES in healthy cells.

In light of the absence of detected EMT, RNAseq data was further examined for indicators of endothelial-to-mesenchymal transition (Hamczyk et al., 2024; Weinstein et al., 2020). Key transcription factors of this process SNAI1/2 and NFATC1 were not found to be upregulated in our dataset. TWIST1 shows slight upregulation after shear stress in HGPS viECs, but is also expressed in very low abundance and thus its effect may be negligeable. Our data therefore does not indicate any shear-stress related endothelial-to-mesenchymal transition in our viECs.

While cholesterol metabolism is crucial for many cellular components, its increased presence in vasculature in the form of oxidative low-density lipoprotein (oxLDL) is a contributor to atherosclerosis (Blanda et al., 2020), making the overrepresentation of the Cholesterol Homeostasis gene set a significant find for HGPS endothelium. LGALS3, encoding galectin-3, is part of this set and is a known mediator of atherosclerosis (Blanda et al., 2020). Recently, the galectins were discovered to be shear-sensitive in the endothelium, with galectin-3 upregulation found in disturbed, atheroprone flow conditions (Lightfoot et al., 2025). Galectin-3 is a pro-inflammatory molecule that drives oxidative stress and the inflammatory response (Blanda et al., 2020). Increased expression is found both in hypertensive patients and in the hypertensive mouse model (Pang et al., 2023) and galectin-3 treatment in endothelial cells leads to monocyte chemotaxis (Blanda et al., 2020) which is the foundation of atherogenesis. Robust increase of galectin-3 in HGPS cells, which is restored when the mutation is corrected, indicates a novel relationship between progerin expression and an increased galectin-3 shear stress response, which can contribute to a pro-inflammatory condition in HGPS vasculature.

Taken together, this study provides evidence that atheroprotective laminar shear stress promotes aberrant endothelial function in HGPS endothelium, including overexpression of LGALS3 which promotes monocyte adhesion and inflammation. This problematic flow response is directly linked to the progerin mutation, and creates the conditions for rapid atherosclerosis development, contributing to the prominence of atherosclerosis in HGPS patients.

## Data Availability

The original contributions presented in the study are publicly available. This data can be found here: https://www.ncbi.nlm.nih.gov/geo/query/acc.cgi?acc=GSE311520.
