# Correction: Hutchinson-Gilford progeria syndrome alters the endothelial genetic response to laminar shear stress

**DOI:** 10.3389/fphys.2026.1902160

**Published:** 2026-06-22

**Authors:** Crystal C. Kennedy, Jonathan L. Carter, George A. Truskey

**Affiliations:** 1 University Program in Genetics and Genomics, Duke University, Durham, NC, United States; 2 Department of Biomedical Engineering, Duke University, Durham, NC, United States

**Keywords:** HGPS: Hutchinson-Gilford progeria syndrome, endothelium, shear stress (fluid), atherosclerosis, LGALS3

A correction has been made to Section 2.2: *Differentiation of iPSCs to endothelial cells (viECs) and viEC culture*. The source of the induced pluripotent stem cells was omitted. The following is the first sentence of the section:

“Three healthy (168CL2, 168CL3, and 0901B) and 2 HGPS (0031D and 167CL2) induced pluripotent stem cells were provided by the Progeria Research Foundation.”

The original version of this article has been updated.

