# Supplementary material for: Hutchinson-Gilford progeria syndrome alters the endothelial genetic response to laminar shear stress
Source: Front Physiol. 2026 Feb 24;16:1599339. doi: 10.3389/fphys.2025.1599339 (PMC12971680; doi:10.3389/fphys.2025.1599339)
Supplement: Supplementary file 1 [file DataSheet1.pdf]

Supplementary Material

Table of Contents

1 Supplementary Figures 2

Supplementary Figure 1. 2

Supplementary Figure 2. 3

Supplementary Figure 3. 4

2 Supplementary Tables 5

Supplementary Table 1. 5

Supplementary Table 2. 6

Supplementary Table 3. 7

1 Supplementary Figures

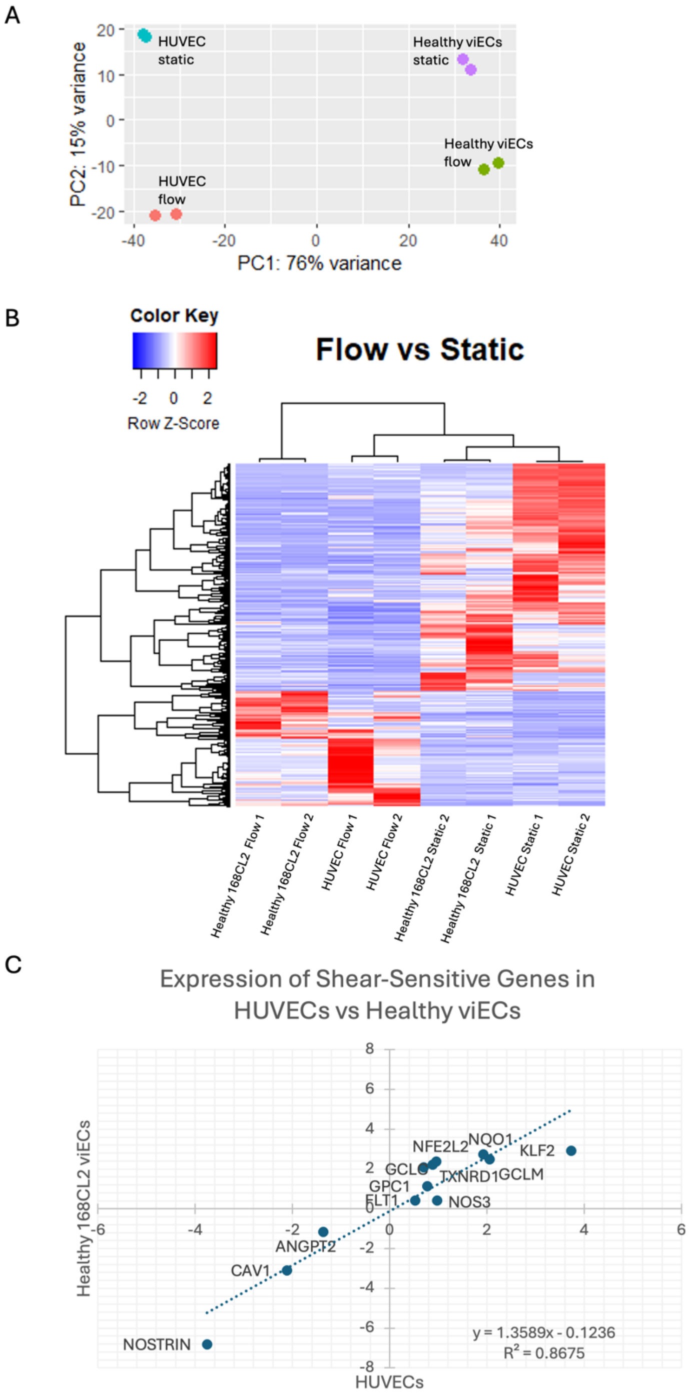

Supplementary Figure 1. Comparison of HUVEC and healthy viEC gene expression in the shear stress response. A) PCA showing healthy and HUVEC flow and static samples. B) Heatmap shows genes with L_2FC of <=|2| and p <= 0.05. C) Differential expression of known shear-sensitive genes shows a high correlation between HUVECs and healthy viECs. Each point represents the L_2FC of gene expression after 24h shear stress at 12dynes/cm^3.

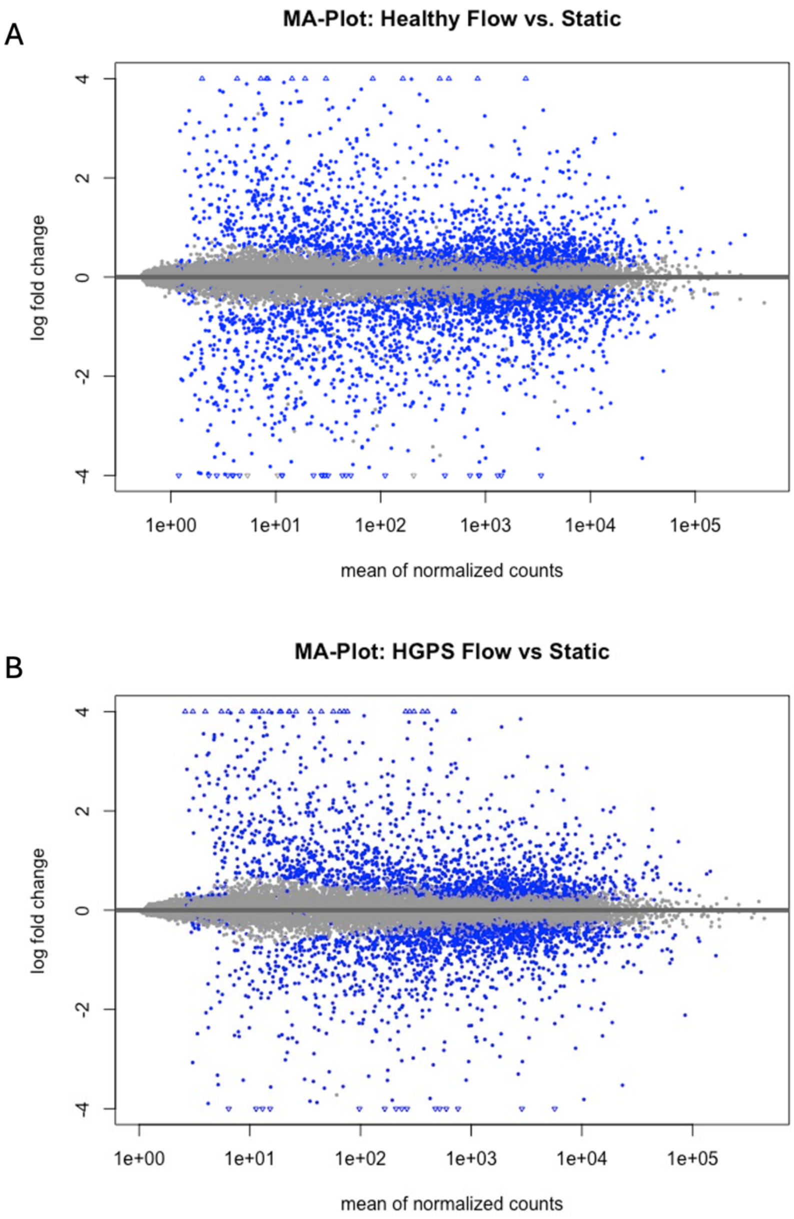

Supplementary Figure 2. MA Plots showing mean of normalized counts vs L2FC in response to flow for A) healthy and B) HGPS viECs. Counts are normalized to static samples and shrunk using the *apeglm* method.

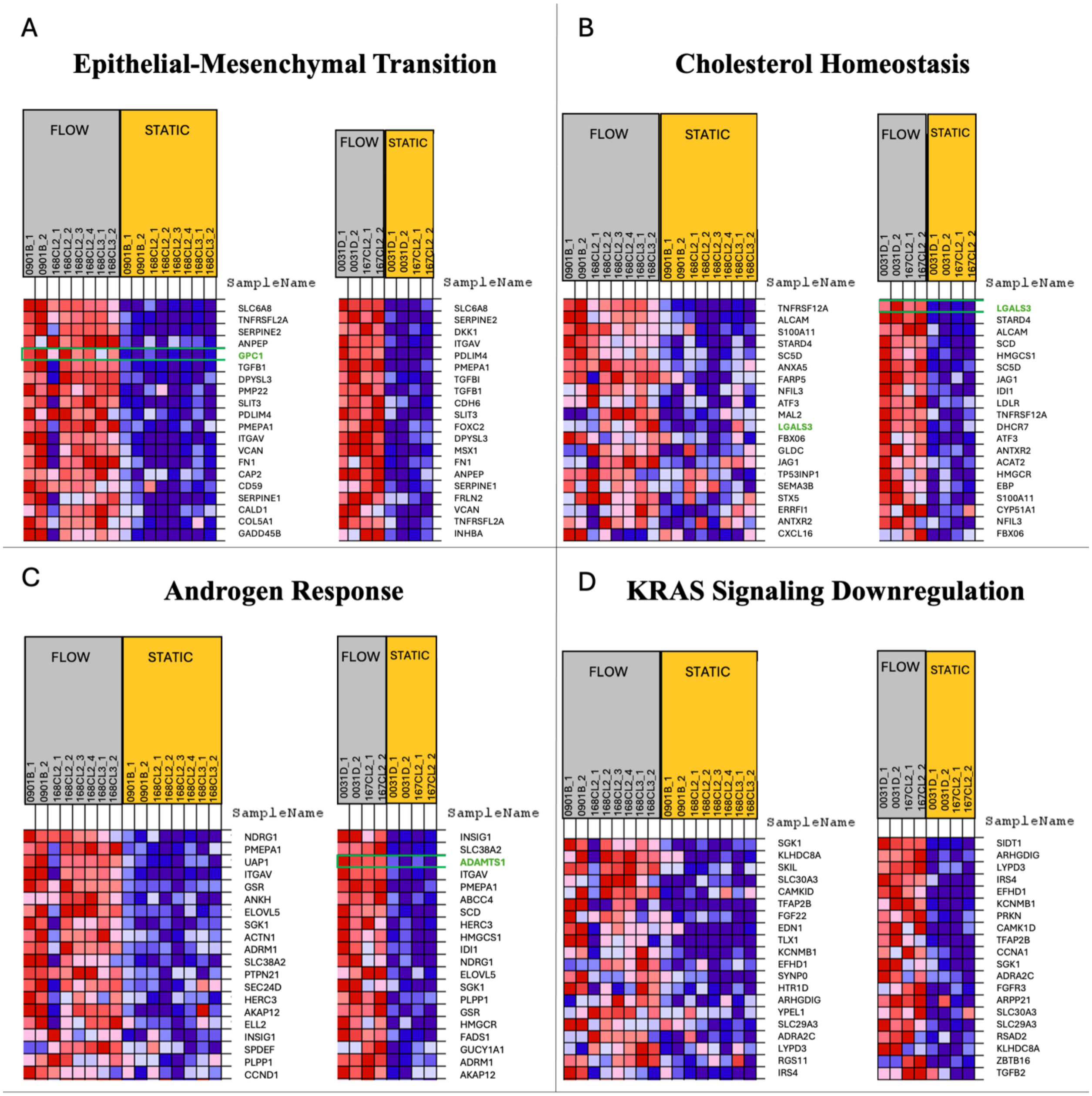

Supplementary Figure 3. Heatmaps of select GSEA results for healthy and HGPS viECs after shear-stress. The top 20 leading edge genes for each phenotype are shown. Select genes of interest are outlined in green.

2 Supplementary Tables

Supplementary Table 1. Quality of RNA for RNAseq experiment comparing healthy viECs and HUVECs

Cell Line	Phenotype	Condition		Q30%
168CL2	Healthy viEC	Static 1	L1	97.6
			L2	97.41
168CL2	Healthy viEC	Static 2	L1	97.73
			L2	97.54
168CL2	Healthy viEC	Flow 1	L1	97.62
			l2	97.42
168CL2	Healthy viEC	Flow 2	L1	97.69
			L2	97.52
HUVEC	Healthy primary	Static 1	L1	97.59
			L2	97.39
HUVEC	Healthy primary	Static 2	L1	97.52
			L2	97.34
HUVEC	Healthy primary	Flow 1	L1	97.78
			L2	97.59
HUVEC	Healthy primary	Flow 2	L1	97.91
			L2	97.73

Supplementary Table 2. Quality of RNA for RNAseq experiment comparing healthy and HGPS viEC shear stress responses. Pair ended sequencing was carried out on all samples.

Date Obtained	Cell Line	Phenotype	Condition	Q30%	
18-Apr-21	0901B	Healthy	Flow 1	*L1*	92.95
				L2	93.09
20-Apr-21	0901B	Healthy	Flow 2	*L1*	93.27
				L2	93.41
18-Apr-21	0901B	Healthy	Static 1	*L1*	93.28
				L2	93.42
20-Apr-21	0901B	Healthy	Static 2	*L1*	93.48
				L2	93.61
29-Jul-20	168CL2	Healthy	Flow 1	*L1*	93.60
				L2	93.76
11-Oct-20	168CL2	Healthy	Flow 2	*L1*	93.36
				L2	93.49
11-May-21	168CL2	Healthy	Flow 3	*L1*	93.45
				L2	93.59
13-May-21	168CL2	Healthy	Flow 4	*L1*	93.28
				L2	93.42
29-Jul-20	168CL2	Healthy	Static 1	*L1*	93.44
				L2	93.59
11-Oct-20	168CL2	Healthy	Static 2	*L1*	93.46
				L2	93.60
11-May-21	168CL2	Healthy	Static 3	*L1*	93.35
				L2	93.49
13-May-21	168CL2	Healthy	Static 4	*L1*	93.25
				L2	93.38
5-Nov-20	168CL3	Healthy	Flow 1	*L1*	93.59
				L2	93.74
10-Nov-20	168CL3	Healthy	Flow 2	*L1*	93.21
				L2	93.35
5-Nov-20	168CL3	Healthy	Static 1	*L1*	93.17
				L2	93.33
10-Nov-20	168CL3	Healthy	Static 2	*L1*	93.08
				L2	93.22
28-Jul-22	0031D	HGPS	Flow 1		93.07
30-Jul-22	0031D	HGPS	Flow 2		93.75
28-Jul-22	0031D	HGPS	Static 1		92.04
30-Jul-22	0031D	HGPS	Static 2		93.20
27-Oct-20	167CL2	HGPS	Flow 1	*L1*	93.45
				L2	93.61
17-Nov-20	167CL2	HGPS	Flow 2	*L1*	93.29
				L2	93.45
27-Oct-20	167CL2	HGPS	Static 1	*L1*	93.14
				L2	93.31
17-Nov-20	167CL2	HGPS	Static 2	*L1*	93.31
				L2	93.45

Supplementary Table 3. Primer sequences used in RT-PCR experiments. All were done at a Tm of 60°C for 40 cycles.

Gene	PrimerBank ID	Sequence (5'-3')		Product size (bp)
GPC1	167001140c1	F -	TGAAGCTGGTCTACTGTGCTC	165
		R -	CCCAGAACTTGTCGGTGATGA	
NOSTRIN	284172476c1	F -	CAGAAAGACACAGCAGCGTTA	238
		R -	CAGAAGATGCCTTGCTCACAATA	
LGALS3	294345474c1	F -	ATGGCAGACAATTTTTCGCTCC	173
		R -	GCCTGTCCAGGATAAGCCC	
ADAMTS1	50845383c3	F -	CAGAGCACTATGACACAGCAA	80
		R -	AGCCATCCCAAGAGTATCACA	
